# Comparative Analysis of Clinical Outcomes for COVID-19 and Influenza among Cardiac Transplant Recipients in the United States

**DOI:** 10.3390/v15081700

**Published:** 2023-08-05

**Authors:** Daniel J. Chavarin, Aniesh Bobba, Monique G. Davis, Margaret A. Roth, Michelle Kasdorf, Adeel Nasrullah, Prabal Chourasia, Karthik Gangu, Sindhu Reddy Avula, Abu Baker Sheikh

**Affiliations:** 1 Department of Internal Medicine, University of New Mexico Health Sciences Center, Albuquerque, NM 87106, USA; 2 Department of Medicine, John H Stronger Hospital, Chicago, IL 60612, USA; anieshbobba@gmail.com; 3 Rush Medical School, Rush University, Chicago, IL 60612, USA; michelle.kasdorf@gmail.com; 4 Division of Pulmonology and Critical Care, Allegheny Health Network, Pittsburgh, PA 15212, USA; 5 Department of Hospital Medicine, Mary Washington Hospital, Fredericksburg, VA 22401, USA; 6 Department of Internal Medicine, University of Kansas Medical Center, Kansas City, KS 66160, USA; kgangu2@kumc.edu; 7 Department of Interventional Cardiology, Division of Cardiology, University of Kansas, Kansas City, KS 66606, USA; sindhu.avula@outlook.com

**Keywords:** COVID-19, influenza, cardiac transplant, mortality, complications, United States, National Inpatient Sample

## Abstract

COVID-19 infections can lead to worse outcomes in an immunocompromised population with multiple comorbidities, e.g., heart transplant patients. We used the National Inpatient Sample database to compare heart transplant outcomes in patients with COVID-19 vs. influenza. A total of 2460 patients were included in this study: heart transplant with COVID-19 (*n* = 1155, 47.0%) and heart transplant with influenza (*n* = 1305, 53.0%) with the primary outcome of in-hospital mortality. In-hospital mortality (*n* = 120) was significantly higher for heart transplant patients infected with COVID-19 compared to those infected with influenza (9.5% vs. 0.8%, adjusted OR: 51.6 [95% CI 4.3–615.9], *p* = 0.002) along with significantly higher rates of mechanical ventilation, acute heart failure, ventricular arrhythmias, and higher mean total hospitalization cost compared to the influenza group. More studies are needed on the role of vaccination and treatment to improve outcomes in this vulnerable population.

## 1. Introduction

Cardiac transplantation has been the standard of therapy for patients with end-stage heart failure and other cardiovascular diseases who develop severe impairment in functional status despite the optimization of medical therapy [1]. According to the International Society for Heart and Lung Transplantation (ISHLT), there have been over 140,000 heart transplants worldwide with an approximate 90.0% one-year survival rate in North America and 80.0% internationally, and a median survival of over 12 years [2]. Over the past several decades, recipient morbidity and mortality have improved due to advancements in selection criteria for recipients and donors, immunosuppressive therapies, and prevention and management of infections [1,3].

Historically, transplant recipients are at increased risk of viral infections despite the above improvements. Multiple mechanisms have been studied that may contribute, including chronic immunosuppressive medications, prolonged viral shedding, hypogammaglobulinemia, and weakened immune response to vaccinations [4,5,6]. Previous studies have shown infection rates as low as 3.0% for influenza in lung transplant populations and up to 18.0% in bone marrow transplant patients [7,8]. Heart transplant recipients are at particular risk of COVID-19 infection and its related complications due to chronic immunosuppression and similar comorbidities that are established risk factors for severe COVID-19 illness (i.e., cardiovascular disease, advanced age, diabetes mellitus, and sex) [9].

Viral illnesses, such as COVID-19, are linked to worse outcomes among cardiac transplant patients [10,11,12]. In the literature, mortality ranges from 24.0 to 26.3% for hospitalized orthotopic heart transplant recipients [10,11,12]; however, large-scale studies evaluating outcomes in heart transplant patients with COVID-19 infection are limited.

Our study aims to assess outcomes between heart transplant patients with COVID-19 and influenza infections utilizing data from the National Inpatient Sample (NIS). The primary outcome was in-hospital mortality. Secondary outcomes were acute kidney disease on hemodialysis, acute heart failure, pulmonary embolism, cerebrovascular accident (CVA), atrial arrhythmia, ventricular arrhythmia, conduction anomalies, sudden cardiac arrest, cardiogenic shock, heart transplant rejection, mean total hospital charge, mean length of hospital stay, and disposition.

## 2. Materials and Methods

This retrospective study utilized the NIS Healthcare Cost Utilization Project (HCUP) sponsored by the Agency for Healthcare and Research and Quality (AHRQ) database, which is an all-payer database that approximates a 20% stratified sample of discharges from US community hospitals [13]. In this analysis, we used the 2016 to 2020 NIS data sets, which included hospitalization from 1 January 2016 to 31 December 2020 and was made available to the public in October 2022.

All patients 18 years of age and older admitted to the hospital who had undergone a cardiac transplant and subsequently contracted COVID-19 or influenza were included in this study. International classification of diseases 10th—clinical modification (ICD-10-CM) codes were used to retrieve patient samples with these conditions, and ICD-10 procedure codes were used to identify inpatient procedures. A detailed code summary is provided in Supplementary Table S1. Patients who were under the age of 18 years, as well as those with missing data points in the variables of interest, were excluded from this study.

### 2.1. Covariates

The NIS database contains data regarding in-hospital outcomes, procedures, and other discharge-related information. Variables were divided into patient-related, hospital-related, and indicators of illness severity as below:

- Patient: age, race, sex, comorbidities, insurance status, mean income in patient’s zip code, and disposition.
- Hospital: location, teaching status, bed size, and region.
- Illness severity: length of stay, mortality, hospitalization cost, Elixhauser comorbidity score.
- In-hospital complications: as below.

### 2.2. Study Outcomes

The primary outcome was in-hospital mortality. Secondary outcomes were acute kidney disease on hemodialysis, acute heart failure, pulmonary embolism, cerebrovascular accident, atrial arrhythmia, ventricular arrhythmia, conduction abnormalities, sudden cardiac arrest, cardiogenic shock, heart transplant rejection, mean total hospital charge, mean length of hospital stay, and disposition.

### 2.3. Statistical Methods

Descriptive statistics were used to summarize the continuous and categorical variables. Continuous variables as mean ± SD; categorical data were summarized as the number and percentage. Univariate analyses for between-group comparisons used the Rao–Scott Chi-square test for categorical variables (e.g., sex and risk factors) and weighted simple linear regression for continuous variables (e.g., age). A multivariate regression model was used to obtain adjusted odds ratio. All analysis was performed using Stata 90 software version 17.0 (Stata Corporation, College Station, TX, USA). *p*-values of less than 0.05 were considered statistically significant.

## 3. Results

### 3.1. Demographics and Baseline Comorbidities

Our study comprised 1155 hospitalized patients who underwent heart transplants and contracted COVID-19 between 1 January and 31 December 2020, along with 1305 similar patients who were infected with influenza from 1 January 2016 to 31 December 2020.

Statistically significant differences were found in a few baseline comorbidities between COVID-19 and influenza cohorts. Notably, the COVID-19 cohort had a significantly higher prevalence of rheumatoid arthritis (3.0% vs. 0.4%, *p* = 0.020), coagulopathy (18.6% vs. 11.9%, *p* = 0.038), obesity (22.5% vs. 11.9%, *p* = 0.001), and chronic kidney disease (CKD) (39.8% vs. 26.8%, *p* = 0.003). Moreover, a significantly higher proportion of these patients was seen at urban teaching hospitals (90.0% and 88.9% for COVID-19 and influenza cohorts, respectively, *p* = 0.030).

Conversely, variations in factors such as gender, age group, ethnicity, income percentile, and insurance type were observed between the two cohorts but were not statistically significant. For instance, both COVID-19 and influenza infections were more common among male heart transplant patients, and both cohorts had a substantial representation from Caucasians. Differences were also observed in the prevalence of hypothyroidism, liver disease, metastatic cancer, solid tumor without metastasis, weight loss, alcohol abuse, drug abuse, and hypertension between COVID-19 and influenza cohorts; however, these were not statistically significant. Table 1 provides a detailed outline of the baseline characteristics of the study cohorts.

### 3.2. In-Hospital Mortality

In-hospital mortality (*n* = 120) was significantly higher for the heart transplant group infected with COVID-19 compared to the influenza group (9.5% vs. 0.8%, respectively, adjusted OR (aOR): 51.6 [95% CI 4.3–615.9], *p* = 0.002) (Table 2).

**Figure 1:**
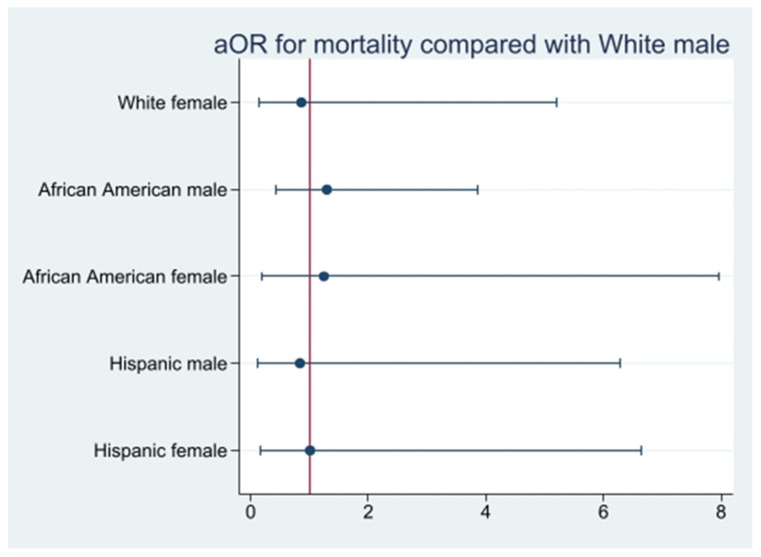
aOR for mortality compared with White males.

A sub-analysis of mortality among White females, Black males, Black females, Hispanic males, and Hispanic females in comparison to White males was performed; however, no statistical significance was found (Table 3 and Figure 1).

### 3.3. In-Hospital Complications

Heart transplant patients infected with COVID-19 had a significantly higher rate of mechanical ventilation use (13.9% vs. 5.4%, aOR: 4.0 [95% CI 1.2–13.1], *p* = 0.021), as well as development of acute heart failure (3.5% vs. 4.6%, aOR: 0.2 [95% CI 0.1–0.9], *p* = 0.031) and ventricular arrhythmias (1.7% vs. 1.5%, aOR: 31.3 [95% CI 1.6–608.5], *p* = 0.023), than those infected with influenza. Rates of acute kidney injury on hemodialysis (6.9% vs. 1.9%, aOR: 3.7 [95% CI 0.7–21.3], *p* = 0.137), pulmonary embolism (1.3% vs. 0.4%, aOR: 1.5 [95% CI 0.0–72.6], *p* = 0.829), CVA (1.3% vs. 0.8%, aOR: 1.1 [95% CI 0.2–6.9], *p* = 0.948), atrial arrhythmias (15.6% vs. 10.7%, aOR: 1.3 [95% CI 0.5–3.4], *p* = 0.576), conduction abnormalities (10.0% vs. 6.9%, aOR: 1.9 [95% CI 0.7–5.1], *p* = 0.231), cardiogenic shock (1.3% vs. 0.8%, aOR: 0.1 [95% CI 0.0–4.4], *p* = 0.201), and heart transplant rejection (1.3% vs. 0.4%, aOR: 7.2 [95% CI 0.9–57.1], *p* = 0.062) were nearly all more prevalent among the COVID-19 cohort compared to the influenza cohort but were not statistically significant (Table 2).

### 3.4. In-Hospital Quality Measures and Disposition

Heart transplant patients infected with COVID-19 had an increased mean length of stay (9.4 days vs. 5.9 days, adjusted length of stay 0.6 days lower, *p* = 0.877) than heart transplant patients infected with influenza. The COVID-19 cohort also had a significantly higher mean total hospitalization charge (USD 138,967 vs. USD 52,803, adjusted total charge USD 84,623 higher, *p* = 0.001). Of those who survived, heart transplant patients with COVID-19 had significantly more discharges to skilled nursing facilities (SNF), long-term acute care (LTAC) facilities, and nursing homes (14.1% vs. 9.6%, *p* = 0.036), as well as discharges to home with home health services (18.8% vs. 11.2%, *p* = 0.036), compared to those with influenza (Table 2).

## 4. Discussion

In this retrospective analysis, we identified 2460 hospitalized patients with known heart transplant, out of which 1155 (47.0%) were diagnosed with COVID-19 infection between 1 January and 31 December 2020, and 1305 (53.0%) were diagnosed with influenza infection between 1 January 2016 and 31 December 2020. Major findings of our study are as follows: (1) COVID-19 patients undergoing heart transplant had significantly increased in-hospital mortality compared to patients infected with influenza. (2) Patients in the COVID-19 cohort also had significantly higher rates of mechanical ventilation use, acute heart failure, ventricular arrhythmias, higher mean total hospitalization cost, and discharges to SNF, LTAC, nursing home, or with home health. (3) The COVID-19 cohort was more likely to have rheumatoid arthritis, coagulopathy, obesity, and CKD.

Influenza has significant mortality in the general population. Annual mean influenza-associated mortality rates for underlying pneumonia and influenza deaths, underlying respiratory and circulatory deaths, and all-cause deaths were 3.1, 13.8, and 19.6 per 100,000 person-years, respectively [14]. The majority of studies analyzing influenza infections in solid organ transplants are in kidney and lung transplants, and there is limited literature specifically on heart transplant patients. Studies looking at isolated heart transplant recipients include case reports of patients with H1N1 influenza with ranging severity from asymptomatic to mechanical ventilation [15,16]. Articles looking at solid organ transplant show substantial morbidity and mortality, with one article reporting admission to hospital in 71.0%, admission to ICU in 16.0%, pneumonia in 32.0%, and death in 4.0% of cases [17]. Additionally, Gainer et al. found that solid organ transplant patients diagnosed with H1N1 had decreased 30-day survival compared to non-transplant patients with H1N1, 83.0% vs. 97.0%, respectively [18]. In Montoya et al., they found that infectious complications in heart transplants are most common with HSV (23.7%), shingles (25.1%), and CMV (14.1%) [19]. They also report that even though their cohort was not routinely vaccinated against influenza, cases were still infrequent [19].

In Diaz-Arocutipa et al. and Singhvi et al., heart transplant recipients were mostly male at 77.0% with an average age of 60 years old, and 63.6% male with an average age of 59 years old, respectively [10,20]. This is consistent with our study’s demographics. Reports from 1992–2012 in the ISHLT database showed that 76.0–81.0% of heart transplant recipients were male, which may explain why there was a higher prevalence in males compared to females in our study [2]. The average age of the patients was 60 years old, which may be due to the fact that patients with chronic comorbidities are more prominent among older populations and were more likely to develop severe COVID-19 [21].

Our results showed a predominance of COVID-19 infection in Caucasian individuals followed by African Americans, and that African Americans and Hispanics with cardiac transplants were more susceptible to COVID-19 infection. In Genuardi et al., 42.0% of COVID-19 cases were among persons who self-identified as Black, and 44.0% of patients identified as White [21]. According to data from the Organ Procurement and Transplantation Network, only 17.0% of heart transplant recipients between 1989 and 2022 were African American [22]. Wolfe et al. looked at racial disparities among heart transplant patients with COVID-19 and reported three times higher mortality in African American and Hispanic races compared to Caucasian after adjustments in social determinants of health and competing risk of non-COVID death [23]. However, they hypothesize that this difference is due to additional social determinants of health not included in their study rather than the biological difference between racial groups [23].

According to our data, transplant patients with COVID-19 were more likely to have rheumatoid arthritis, coagulopathy, chronic kidney disease, and obesity. We also found that there was a higher prevalence of patients with hypothyroidism, hypertension, liver disease, metastatic cancer, solid tumor without metastasis, weight loss, and alcohol and drug abuse. In Bottio et al., the three most common pre-existing conditions were hypertension (66.0%), diabetes mellitus (30.0%), and ischemic heart disease (28.0%) [24]. This is supported by Diaz-Arocutipa et al., who found that hypertension (69.0%), diabetes (36.0%) and chronic kidney disease (36.0%) were the most common comorbidities [20]. In a meta-analysis by Li et al., severe cases and those requiring ICU admission that had diabetes were twice as frequent, which may be due to chronic hyperglycemia and inflammation causing abnormal immune responses [25,26]. One explanation for the diffuse organ complications seen in COVID-19 is that there is a greater expression of ACE2 in patients with cardiovascular disease and other comorbidities which may lead to increased susceptibility [27].

Our findings showed an in-hospital mortality rate of 9.5% in the COVID-19 cohort, which was significantly higher than the influenza control group at 0.8%. In Singhvi et al., mortality was 26.3% in hospitalized COVID-19 patients with orthotopic heart transplants, while additional studies have shown mortalities of 25.0% and 24.0% in hospitalized orthotopic heart transplant and solid organ transplant recipients, respectively [10,11,12]. The differences in outcome between our study and the aforementioned studies are most likely attributable to larger sample sizes or disease severity, factors that are not addressed within our study and are thus a limitation. Recent studies have investigated why there is an increase in mortality in COVID-19; one study has shown that a significant number of healthy patients lacked neutralizing antibodies, which are thought to increase the clearance of the virus, after recovering from COVID-19 infection [28]. These neutralizing antibodies may be even lower in transplant patients given their chronic immunosuppression.

In our study, we found a significant increase in mechanical ventilation, ventricular arrhythmias, and acute heart failure. The literature demonstrates a range of complications, with one study of heart transplant recipients reporting that 50.0% of non-dialysis patients developed acute kidney injury, 17.0% required renal replacement therapy, and 32.0% required mechanical ventilation, and reporting an estimated prevalence of venous thromboembolism at 3.0% and only one patient with new-onset heart failure [21]. In Marcondes-Braga et al., there was an increase in ventricular arrhythmia at 12.5% and an increase in allograft rejection reported at 10.0% [29]. Bottio et al.’s data showed that older age, diabetes mellitus, extracardiac arteriopathy, previous PCI, CAV score, lower GFR, and higher NYHA functional classes were all significantly associated with in-hospital mortality [24]. Additionally, a nationwide survey of all heart transplant centers in Germany examined the outcomes of heart transplant recipients infected with COVID-19 during the first months of the pandemic in Germany [9]. Their study found that high mortality (87.5%) was associated with right ventricular dysfunction (62.5% vs. 7.7%, *p* = 0.014), arrhythmias (50.0% vs. 0.0%, *p* = 0.012), thromboembolic events (50.0% vs. 0.0%, *p* = 0.012), and elevated high-sensitivity cardiac troponin T (hs-cTnT) and N-terminal pro brain natriuretic peptide (NT-proBNP) (*p* = 0.017) [9]. Of those in their study, 38.1% displayed a severe course requiring invasive mechanical ventilation [9]. Piroth et al. found that in-hospital mortality was higher in patients with COVID-19 than in patients with influenza, 16.9% vs. 5.8%, respectively, with a relative risk of death of 2.9 (95% CI 2.8–3.0) [30]. Additionally, Ludwig et al. reported a higher proportion of cases with ICU admission (21.0% vs. 13.0%), mechanical ventilation (15.0% vs. 9.0%), and severe disease (28.0% vs. 16.0%) in COVID-19 patients compared to influenza [31]. The literature is unclear on the cause of the increased complications from COVID compared to influenza. It is noteworthy that the mechanisms of how COVID-19 and other viruses, such as influenza, impact heart transplant patients may differ. For example, COVID-19 directly binds to ACE2 receptors on the surface of the heart, which may contribute to more severe outcomes in some cases [10]. Furthermore, the high incidence of thromboembolic symptoms in COVID-19 patients is likely due to a combination of disseminated intravascular coagulation and myocardial injury [9]. Some articles speculate that it could be due to the systemic cytokine storm causing tissue injury or organ “cross-talk” [32,33]. One area that was not analyzed in our study was the significance of superimposed bacterial infection. One study noted that bacterial nosocomial infections were a leading cause of death in the observed patient population [29]. Notably, in our study, we found an increase in acute kidney injury on hemodialysis, pulmonary embolism, cerebrovascular accident, atrial arrhythmia, conduction abnormalities, cardiogenic shock, and heart transplant rejection; however, the increases were not statistically significant. One reason for poor prognosis in heart transplant patients may be the high burden of comorbidities as patients in the critical/mortal group had a statistically higher proportion of diabetes, cardiovascular disease, and respiratory disease when compared to non-critical populations [34,35].

We found that hospitalization costs were significantly higher in the COVID-19 group. It is important to be aware of the significant increase in discharge to SNF or home health in our heart transplant COVID-19 cohort as studies have shown that readmissions happen more often in patients discharged to SNF or with home health care than discharged to home or with self-care [36].

Studies have investigated theories of cytokine storm contributing to tissue injury in COVID-19. Huang et al. report that pro-inflammatory cytokine concentrations were higher in ICU/non-ICU adults compared to healthy adults [32]. Given that transplant patients are undergoing chronic immunosuppression therapy, there were early theories that this may be protective against the cytokine storm. However, Akama-Garren et al. showed more severe cases of COVID-19 in patients who were on prior immunosuppressive therapy [37]. Additionally, another study did not find evidence of a protective effect of prednisone therapy in their cohort [21].

Despite the decrease in immune response, influenza vaccinations are still recommended in all transplant patients, if not received prior to transplantation [38]. Peled et al. analyzed COVID-19 antibody responses in vaccinated heart transplant recipients and showed that there was a presence of IgG anti-RBD antibodies 21 days after the second dose [39]. It was noted that the majority of systemic adverse events (AE) were mild or moderate without emergency department visits or hospitalizations and transplant patients had significantly lower AE compared to the general population [39]. Some detection of antibodies after the second dose may indicate that there is some protective immunity; however, it is recommended to complete a three-dose series with the appropriate booster.

The ISHLT, American Society of Transplantation, and American Society of Transplant Surgeons published a joint statement regarding COVID-19 vaccination and recommended that all eligible transplant recipients and household members are vaccinated, preferably with the three-dose series [39]. If vaccination is received before transplantation, it is recommended to complete the series a minimum of two weeks prior and if received after transplantation, as early as 1–3 months after [39]. Given viral variants, the study of immune response in immunocompromised populations and boosting the efficiency of vaccines are still areas being explored.

Adult organ transplant recipients experience both short- and long-term influenza-related complications. Vichez et al. found that among hospitalized solid transplant patients with influenza infection, 47.0% developed lower respiratory tract infections and 17.0% developed bacterial pneumonia, and they observed myocarditis, myositis, and bronchiolitis obliterans [40]. They also reported rejection in 61.0% of lung transplant recipients and 100% of kidney transplant recipients [40]. There are reports of lung transplant patients with acute (56.0%) and chronic (11.0%) graft rejection after influenza infections [41]. In pediatric solid organ transplant patients, Mauch et al. followed five cases of influenza infection in the first year following transplant and reported two cases of severe respiratory and central nervous system complications [42]. Given the new phenomenon of long COVID, there are concerns regarding long-term complications of COVID-19 in immunosuppressed populations as well. While there are limited data on long-term complications in cardiac transplant recipients in particular, one study in the UK looked at long-term complications in the general population and showed at median follow-up of 5.9 months that only 239 (28.8%) of 830 participants felt fully recovered, 158 (19.6%) of 806 had a new disability (assessed by the Washington Group Short Set on Functioning), and 124 (19.3%) of 641 experienced a health-related change in occupation [43]. There is one study in pediatric heart transplant patients that reported that 96.0% of candidates and recipients had resolution of their symptoms at 30 days, 3.0% had an unresolved course, and 1.0% of the patients were reported to have significant long-term sequelae [44]. Future studies should focus on additional long-term complications, especially in vulnerable populations such as immunosuppressed adult patients.

## 5. Future Implications

Our study is based on the NIS 2020 database and includes primarily non-vaccinated patients, as the FDA granted the first vaccinations against COVID-19 emergency use authorization in December 2020 in the USA. With higher morbidity and mortality noted in our study in cardiac transplant patients with COVID-19, our study may serve as an important lesson and valuable reference for non-vaccinated cardiac transplant patients, especially regarding vaccine hesitancy. We could not study the effects of vaccination and long COVID in cardiac transplant patients with COVID-19. These should be the focus of future studies to improve the COVID-19-related outcome in this immunocompromised population.

## 6. Limitations

Our investigation, while offering valuable insights, is subject to several constraints that need to be acknowledged when interpreting our results. Chief among these is the retrospective design of our study, which potentially exposes our findings to selection bias. Data for our research was derived from the NIS database. While this database provides an extensive array of information, it does not incorporate specific parameters such as laboratory results, imaging data, and key transplant-related variables such as donor-recipient size and gender mismatch, panel reactive antibodies, and the use of ancillary support at the time of implantation such as ventilators, extracorporeal membrane oxygenation, and ventricular assist devices. As a result, diagnoses of cardiac transplantation and the accompanying comorbidities, all of which rely on ICD-10 codes, may be prone to potential inaccuracies. Furthermore, our cohort predominantly consists of patients diagnosed in urban teaching institutions, which are typically furnished with more comprehensive resources compared to smaller, non-urban facilities. This discrepancy might constrain the broad applicability of our results to other healthcare settings that differ in their geographical location or institutional size. With respect to the viral infections studied, constraints associated with ICD-10 coding limited our differentiation to influenza A and other forms of influenza. Similarly, the NIS database does not permit us to ascertain the vaccination status for either influenza or COVID-19, which could introduce a potential source of bias into our comparative analysis. Additionally, due to lack of granular data, we were unable to account for the potential impact of other respiratory pathogens, including antibiotic-resistant bacteria and other respiratory viruses such as RSV, CMV, parainfluenza, and human metapneumovirus. Lastly, the temporal parameters of our study mean that it primarily encompasses individuals who were non-vaccinated, given that our study timeline largely predates the first approval of the COVID-19 vaccination under an emergency use authorization (EUA) by the FDA on 11 December 2020.

Despite these constraints, the robust sample size of our study lends significant weight to our findings. This investigation serves as an important stepping-stone for future research, which should strive to incorporate these limitations for a more exhaustive understanding of clinical outcomes among cardiac transplant recipients confronting COVID-19 and influenza infections.

## 7. Conclusions

Our study utilizing National Inpatient Sample 2020 found increased in-hospital mortality and other complications, including higher rates of mechanical ventilation, acute heart failure, and ventricular arrhythmias in patients with COVID-19 compared to patients with influenza in cardiac transplant recipients. Vaccination may play a role in reducing COVID-19-related morbidity and mortality in cardiac transplant recipients. Given limited evidence regarding COVID-19-related outcomes and prevention and treatment strategies in cardiac transplant recipients, further research should focus on these areas to improve outcomes in this vulnerable population.

## Figures and Tables

**Table 1:** Heart transplant patients with COVID-19 and influenza unmatched patient-level characteristics.

Characteristics	Heart Transplant Patients with COVID-19	Heart Transplant Patients with Influenza	*p*-Value
*n* = 2460	*n* = 1155 (47.0%)	*n* = 1305 (53.0%)	
Sex (Female)	24.2%	23.4%	0.828
Mean age years (SD)			0.249
Male	61.3 (14.2)	59.8 (14.8)	
Female	57.4 (14.4)	54.9 (15.5)	
Age Groups			0.150
≥18–29	3.9%	7.3%	
30–49	17.3%	15.3%	
50–69	48.5%	54.0%	
≥70	30.3%	23.4%	
Race			0.282
Caucasians	49.3%	55.1%	
African American	31.7%	25.6%	
Hispanics	15.9%	13.0%	
Asian or Pacific Islander	0.9%	3.2%	
Native American	0.4%	1.2%	
Others	1.8%	2.0%	
Median Household Income			0.075
0–25th percentile	27.1%	29.3%	
26–50th percentile	30.1%	21.1%	
51–75th percentile	26.2%	26.2%	
≥76th percentile	16.6%	23.4%	
Insurance Status			0.521
Medicare	65.3%	63.0%	
Medicaid	9.8%	7.0%	
Private	24.0%	28.8%	
Self-pay	0.9%	1.2%	
Hospital Division			0.753
New England	2.6%	5.8%	
Middle Atlantic	19.1%	13.8%	
East North Central	14.7%	17.6%	
West North Central	9.1%	7.7%	
South Atlantic	16.5%	20.3%	
East South Central	6.9%	6.5%	
West South Central	15.6%	12.6%	
Mountain	6.1%	5.4%	
Pacific	9.5%	10.3%	
Hospital Bedsize			0.960
Small	9.1%	8.4%	
Medium	17.3%	18.0%	
Large	73.6%	73.6%	
Hosptal Teaching Status			0.030
Rural	5.6%	2.3%	
Urban non-teaching	4.3%	8.8%	
Urban teaching	90.0%	88.9%	
Comorbidities Mean Elixhauser Score (SD)	4.8 (1.9)	4.2 (1.9)	
Pulmonary Circulation Disorder	5.6%	6.1%	0.816
Peripheral Vascular Disorder	13.4%	14.6%	0.714
Hypertension	64.5%	60.5%	0.360
Chronic Pulmonary Disease	16.9%	23.0%	0.094
Diabetes	53.7%	54.0%	0.930
Hypothyroidism	18.2%	16.1%	0.533
Liver Disease	6.5%	4.2%	0.262
Lymphoma	0.9%	1.5%	0.502
Metastatic Cancer	1.3%	0.4%	0.295
Solid Tumor without Metastasis	3.5%	1.5%	0.185
Rheumatoid Arthritis	3.0%	0.4%	0.020
Coagulopathy	18.6%	11.9%	0.038
Obesity	22.5%	11.9%	0.001
Weight Loss	10.0%	8.1%	0.465
Alcohol Abuse	2.2%	0.4%	0.077
Drug Abuse	1.7%	1.5%	0.862
Depression	14.7%	17.6%	0.408
Chronic Kidney Disease	39.8%	26.8%	0.003

**Table 2:** In-hospital outcomes for heart transplant patients with COVID-19 and influenza.

Variable	Heart Transplant Patients with COVID-19	Heart Transplant Patients with Influenza	*p*-Value
In-hospital mortality (*n* = 120)	9.5%	0.8%	
	Adjusted odds ratio ^1^ 51.6 (4.3–615.9)		0.002
Mechanical ventilation	13.9%	5.4%	
	Adjusted odds ratio ^1^ 4.0 (1.2–13.1)		0.021
Acute kidney injury on hemodialysis	6.9%	1.9%	
	Adjusted odds ratio ^1^ 3.7 (0.7–21.3)		0.137
Acute heart failure	3.5%	4.6%	
	Adjusted odds ratio ^1^ 0.2 (0.1–0.9)		0.031
Pulmonary embolism	1.3%	0.4%	
	Adjusted odds ratio ^2^ 1.5 (0.0–72.6)		0.829
Cerebrovascular accident	1.3%	0.8%	
	Adjusted odds ratio ^1^ 1.1 (0.2–6.9)		0.948
Atrial arrhythmia	15.6%	10.7%	
	Adjusted odds ratio ^1^ 1.3 (0.5–3.4)		0.576
Ventricular arrhythmias	1.7%	1.5%	
	Adjusted odds ratio ^1^ 31.3 (1.6–608.5)		0.023
Conduction abnormalities	10.0%	6.9%	
	Adjusted odds ratio ^1^ 1.9 (0.7–5.1)		0.231
Sudden cardiac arrest	0.4%	0.8%	
	Adjusted odds ratio ^2^ 1.4 (0.1–24.1)		0.809
Cardiogenic shock	1.3%	0.8%	
	Adjusted odds ratio ^2^ 0.1 (0.0–4.4)		0.201
Heart transplant rejection	1.3%	0.4%	
	Adjusted odds ratio ^3^ 7.2 (0.9–57.1)		0.062
Myocardities	0.43%	0.38	
	Adjusted odds ratio ^3^ 0.83 (0.07–10.1)		0.08
Mean total hospitalization charge (USD)	138,967	52,803	
	84,623 higher		0.001
Mean length of stay (days)	9.4	5.9	
	0.6 days lower		0.877
Disposition			0.036
Home/Routine	67.2%	78.8%	
SNF ^4^/LTAC ^5^/Nursing home	14.1%	9.6%	
Home health	18.8%	11.2%	
AMA ^6^	-	0.4%	

^1^ Adjusted for age, sex, race, insurance status, discharge quarter, Elixhauser co-morbidities, hospital location, teaching status, and bed size; ^2^ Adjusted for age, sex, race, weekend admission, insurance status, Elixhauser co-morbidities; ^3^ Adjusted for age, sex, Elixhauser co-morbidities; ^4^ Skilled nursing facility; ^5^ Long-term acute care facility; ^6^ Against medical advice.

**Table 3:** aOR for mortality when compared to White males.

	aOR	95% CI (LL-UL)	*p*-Value
White female	0.9	0.1-5.2	0.870
Black male	1.3	0.4-3.9	0.630
Black female	1.2	0.2-8.0	0.810
Hispanic male	0.8	0.1-6.3	0.860
Hispanic female	1.0	0.2-6.6	0.980

## Data Availability

The data used in this study are from the National (Nationwide) Inpatient Sample for the year 2020, obtained from the Healthcare Cost and Utilization Project. The NIS is a publicly available database, and researchers interested in accessing the data can obtain it directly from the HCUP website (https://www.hcup-us.ahrq.gov/; accessed on 18 March 2023).

## Supplementary Materials

The following supporting information can be downloaded at: https://www.mdpi.com/article/10.3390/v15081700/s1, Table S1: ICD-10 codes.
