# Supplementary material for: Comparative Analysis of Clinical Outcomes for COVID-19 and Influenza among Cardiac Transplant Recipients in the United States
Source: Viruses. 2023 Aug 5;15(8):1700. doi: 10.3390/v15081700 (PMC10458639; doi:10.3390/v15081700)
Supplement: Supplementary file 1 [file viruses-15-01700-s001.zip › viruses-2531481-supplementary.pdf]

Table S1

Diagnosis	ICD-10 code
heart transplant	Z941,Z4821
Influenza	J10,J11,J09
Covid 19	U071, U00, U49, U50, U85, J1282
Acute Heart Failure	I5021,I5023,I5031,I5033,I5041,I5043,I5043,I50811,I50813,I50814
Pulmonary embolism	I2602, I2609, I2692, I2693, I2694, I2699
Acute CVA	I63
Atrial Arrhythmia	I48 I480 I481 I4811 I4819 I482 I4820 I4821 I483 I484 I489 I4891 I471
Ventricular Arrhythmia	I4901 I4902 I472 I4720 I4721 I4729 I470
Conduction Abnormalities	I495 I440 I441 I442 I4430 I4439 I444 I445 I4460 I4469 I447 I450 I4510 I4519 I452 I453 I454 I455 I456 I458 I4581 I4589 I459
STEMI	I210,I211,I212,I213,I219
NSTEMI	I214
Heart transplant rejection	T8621
Mechanical ventilation	5A1945Z,5A1955Z,5A1935Z,5A09357,5A09457,5A09557
vasopressor	3E030XZ,3E033XZ,3E040XZ,3E043XZ,3E050XZ,3E053XZ,3E060XZ,3E063XZ
Sudden cardiac arrest	I46, I97
AKI and hemodialysis	N17, N990,5A1D70Z,5A1D90Z,5A1D80Z,5A1D00Z,5A1D60Z
Cardiogenic Shock	R570
Mechanical Circulatory Support	5A02110, 5A02210, 5A0211D, 02HA3RZ, 5A02116, 5A0221D, 5A1522F, 5A1522G, 5A1522H, 5A15A2F, 5A15A2G, 5A15A2H, 5A15223
CKD	N181, N182, N1830, N1831, N1832, N184,N185,N189
Pulmonary Circulation Disorder	Data Obtained from elixhauser comorbidity index
Chronic Pulmonary Disease	
Diabetes Uncomplicated	
Diabetes Complicated	
Hypothyroidism	
Peripheral vascular disorder	
Depression	
coagulopathy	
Weight loss	
Peptic Ulcer Disease (excluding bleeding)	
Lymphoma	
Metastatic Cancer	
Solid Tumor Without Metastasis	
Rheumatoid Arthritis/Collagen Vascular	

Obesity	
Drug Abuse	
HTN	
PAD	
OSA	
Liver Disease	
Alcohol	
Smoking	F17, F172, F1720, F17200, F17201, F17203, F17208, F17209, F1721, F17210, F17211, F17213, F17218, F17219, F1722, F17220, F17221, F17223, F17228, F17229, F1729, F17290, F17291, F17293, F17298, F17299, Z87891
Hx of PCI	Z986, Z9861, Z9862
Hx of CABG	Z951
Previous MI	I252
CAD	I2510, I25111, I25118, I25119, I252, I253, I254, I2541, I2542, I255, I256, I257, I2570, I25700, I25701, I25708, I25709, I2571, I25710, I25711, I25718, I25719, I2572, I25720, I25721, I25728, I25729, I2573, I25730, I25731, I25738, I25739, I2575, I25750, I25751, I25758, I25759, I2576, I25760, I25761, I25768, I25769, I2579, I25790, I25791, I25798, I25799, I258, I2581, I25810, I25811, I25812, I2582, I2583, I2584, I2589, I259
